# Human Papillomavirus and Squamous Cell Carcinoma of Unknown Primary in the Head and Neck Region: A Comprehensive Review on Clinical Implications

**DOI:** 10.3390/v13071297

**Published:** 2021-07-02

**Authors:** Mikkel Hjordt Holm Larsen, Hani Ibrahim Channir, Christian von Buchwald

**Affiliations:** Department of Otorhinolaryngology, Head and Neck Surgery & Audiology, Rigshospitalet, Copenhagen University Hospital, 2100 Copenhagen, Denmark; Hani.Ibrahim.Channir.02@regionh.dk (H.I.C.); christian.von.buchwald@regionh.dk (C.v.B.)

**Keywords:** human papillomavirus, head and neck cancer, unknown primary, cervical lymph node metastasis

## Abstract

Squamous cell carcinoma of unknown primary (SCCUP) is a challenging diagnostic subgroup of oropharyngeal squamous cell carcinoma (OPSCC). The incidence of SCCUP is increasing in parallel with the well-documented increase in OPSCC and is likewise driven by the increase in human papillomavirus (HPV). The SCCUP patient often presents with a cystic lymph node metastasis and undergoes an aggressive diagnostic and treatment program. Detection of HPV in cytologic specimens indicates an oropharyngeal primary tumor origin and can guide the further diagnostic strategy. Advances in diagnostic modalities, e.g., transoral robotic surgery and transoral laser microsurgery, have increased the successful identification of the primary tumor site in HPV-induced SCCUP, and this harbors a potential for de-escalation treatment and increased survival. This review provides an overview of HPV-induced SCCUP, diagnostic modalities, and treatment options.

## 1. Introduction

Annually, more than 90,000 people are diagnosed with oropharyngeal squamous cell carcinoma (OPSCC) worldwide [1]. A subgroup of OPSCC is seen among patients referred with a cervical lymph node metastasis as the primary symptom of malignant disease and where the primary tumor site cannot be identified, hence named squamous cell carcinoma of unknown primary (SCCUP). Patients with SCCUP are estimated to represent up to 10% of the OPSCC cases annually [2]. In parallel with the sharp increase in the incidence of OPSCC, there has been an escalation of patients with SCCUP which is reported to be linked to the significant increase in human papillomavirus (HPV)-induced OPSCC [3,4,5,6]. The diagnostic workup of SCCUP is laborious and often includes a clinical examination and radiologic imaging, i.e., whole body positron emission tomography–computed tomography (PET-CT) or magnetic resonance imaging (MRI) [7,8,9]. If the primary tumor site is not identified, the patient is hereafter referred to pan-endoscopy under general anesthesia with concurrent random or targeted biopsies from the base of the tongue, bilateral palatine tonsillectomy and adenoidectomy, and, in some institutions, transoral robotic surgery (TORS)- or transoral laser microsurgery (TLM)-assisted lingual mucosectomy [7,8,9]. However, the diagnostic algorithm is diverse and remains controversial across institutions and countries, with no uniform consensus and conflicting treatment strategies, as demonstrated by a Nordic survey [10]. If the diagnostic workup fails to identify the primary tumor site, the patient is diagnosed with and subsequently treated for SCCUP. The current treatment approach at the early N-site stage is neck dissection, if deemed technically possible, or definitive radiotherapy (RT) [7,8,11]. With an advanced cancer stage (i.e., solely based on cN/pN), patients are referred to the traditional first-line treatment with (chemo)radiotherapy (CRT) [7,8,11] which often involves wide-field bilateral IMRT radiotherapy of the neck with total mucosal irradiation [8,11,12]. This may lead to a successive quality of life (QoL) decline in domains such as xerostomia, dysphagia, and chewing ability, as demonstrated among OPSCC patients [13,14,15,16].

In parallel with the increasing numbers of SCCUPs induced by HPV, HPV testing of the cervical lymph node metastasis is increasingly applied as a part of the diagnostic workup [17]. HPV detection substantiates the suspicion of OPSCC located in the palatine or lingual tonsils [18] and may further guide the diagnostic workup and treatment and serve as an essential prognostic tool [19].

The aim of this review is to provide an overview of HPV-induced SCCUP, including diagnostic workup strategies for the primary tumor and different treatment options in HPV-induced SCCUP patients.

## 2. Development of HPV SCCUP

HPV is a widespread sexually transmitted disease, e.g., it is the most common sexually transmitted disease in the United States [20], and in the case of OPSCC, it is thought to transmit through genito-oral and oral–anal sexual contact [21]. In the western world, OPSCCs are reported to be caused by HPV in the majority of patients, and there was an upsurge of HPV-induced OPSCC in the United States from 16% in the 1980s to more than 70% during the 2000s [22]. The high incidence of HPV-induced OPSCC is also seen in a number of European countries, with an increase from 35% before 2000 to 73% after 2005 [23], whereas HPV positivity in Asia is reported to occur in 20–34% of OPSCCs [24,25,26,27] with an increasing incidence [24,25,27].

A substantial number of primary tumors identified in SCCUP patients are found in the oropharynx and are HPV-induced, with HPV16 being the predominant high-risk subtype [23,28,29]. The demographic and clinical traits of OPSCC and SCCUP are quite similar. The majority of HPV-positive (+) patients are often males, younger than HPV-negative (-) patients, and tend to exhibit less alcohol and tobacco abuse [5,6,30], which are common causes of HPV disease [31,32]. Both groups often exhibit a cystic lymph node metastasis and an early T-stage, which are also the instances when the primary tumor site is identified in SCCUP [6,33,34,35,36]. However, what constitutes the biological differences between SCCUP and OPSCC and causes a small, undetectable primary tumor or regressed tumor to metastasize early to the regional lymph nodes is unknown and still debatable.

HPV is a double stranded DNA virus that integrates with the host genome in the cell nuclei where transcription of E6 and E7 carcinogenic proteins is activated [37,38]. The transcription of viral carcinogenic proteins, e.g., E6 and E7, in the tumor cell nuclei causes carcinogenesis by interaction with and degradation of the host protein, p53, and inactivation of retinoblastoma protein, both major tumor-suppressor proteins [37,38,39]. This leads to an overexpression of p16, a cyclin-dependent kinase inhibitor often used as a surrogate marker for HPV-positivity [7,8,39]. This overexpression is reflected by the latest update of the 8th edition of the AJCC cancer staging manual in which staging of the cancer is based upon p16 status due to the excellent treatment response and better prognosis in HPV+ patients [40,41]. It is believed that a persistent infection with HPV is the key component of carcinogenesis and the development into OPSCC [38], which has been well described in HPV-induced cervical cancer with HPV16 being the predominant subtype [20]. One study analyzed antibodies against HPV16 in serum samples stored for almost 10 years before OPSCC was diagnosed and demonstrated a 14-fold increase in the risk of developing OPSCC among those displaying seropositivity [42].

Within the oropharynx, a specialized reticulated lymphoepithelial mucosal tissue is found in the crypts of the palatine and lingual tonsils, which are particularly susceptible to HPV infection [28] and take part in immune response initiation [37,43]. The epithelium is also characterized by intraepithelial blood vessels and a discontinuous basal membrane, which could allow for the early metastasis tendency from even small occult tumors to the cervical lymph nodes [28,44]. An immune-mediated response has also been proposed to cause tumor regression [45] and is supported by in vivo and in vitro models reflecting the antitumor and tumor clearance response from CD4+ and CD8+ cells [46]. High counts of CD8+ tumor-infiltrating lymphocytes have likewise been shown to possess a favorable prognostic outcome in patients [47,48,49], emphasizing the possible immune-mediated component in tumor regulation [48,49].

In contrast to HPV, tobacco use causes direct DNA damage and formation of oncogenic DNA adducts that can be traced in human tissue, as also demonstrated in head and neck cancer [50]. Smoking also causes double stranded DNA breaks in the cell, and the inability to repair these DNA damages has been associated with squamous cell carcinoma development [51].

## 3. Identification of the Primary Tumor Site Addressing HPV

A rigorous and aggressive diagnostic workup program is normally accomplished in order to successfully identify the primary tumor and includes thorough physical examination, fine needle aspiration (FNA), radiologic examinations, and examination under general anesthesia [7,8,11].

### 3.1. Fine Needle Aspiration

As patients with HPV+ SCCUP often present with an enlarged cervical lymph node, it is imperative to discriminate between underlying malignancy and benign conditions, e.g., branchial cleft cysts. This evaluation can be performed using an FNA from the lymph node, since radiologic imaging is insufficient in distinguishing between a branchial cleft cyst and the cystic metastasis from HPV+ SCCUP [52]. FNA has been proven as a cost-effective diagnostic tool and a suitable method for molecular testing on preserved DNA [53], and there is no significant difference in the diagnostic rates of cancers such as squamous cell carcinoma, adenocarcinoma, or thyroid carcinoma when compared to core needle sampling [54]. Several HPV testing assays have been validated in cytology specimens and have been shown to be feasible and effective in guiding the diagnostic workup of patients with SCCUP [18,36,55,56,57,58,59]. In addition, FNA is a more reliable tool for identifying the primary in comparison with PET-CT [60]. If HPV is detected in the FNA smear, it substantiates the suspicion of oropharyngeal primary tumor origin [36,58]. HPV–DNA testing can be performed by PCR with a sensitivity of 95% and specificity of 100% or in situ hybridization with a sensitivity and specificity of 91% and 94%, respectively [18,29], with an excellent concordance between HPV status of the cervical metastasis and identified primary tumors [12]. p16-positive (+) immunocytochemistry has similarly been proposed as a surrogate marker for HPV+ with a reasonable concordance with HPV-DNA/mRNA detection, but the technique has a notable inferior specificity [29,61] and is currently widely debated in terms of determining a cutoff criterion for p16 staining [18]. The technique additionally carries a risk of false positivity in benign conditions, e.g., branchial cleft cysts [43,62]. An estimated 10–15% of OPSCC and SCCUP exhibit p16+ but HPV–DNA negativity [35,63,64], and p16 should, therefore, not be used as a basis for de-escalation treatment decisions. This is supported by large cohort studies proving that solely the combination of HPV+ and p16+, compared to p16+ alone, warrants improved survival and minimized risk of distant metastasis [35,65].

### 3.2. Radiological Examination

The radiological examinations used are typically CT or MRI, both providing identical reported primary tumor identification rates of 9–23% [17,60], disregarding HPV status, with MRI being superior in determining extracapsular nodal extension (ECE) [17]. PET-CT is reported as giving identification rates of 29–44% [60,66] and as having a sensitivity and a specificity of 73–97% and 44–68%, respectively [66,67], but with no information on HPV status correlation. A study from the Netherlands (*n* = 31) found that there was no significant association between p16 and HPV status and imaging results [68]. PET-CT has a high false-positive rate due to the physiological uptake of the tracer in normal lymphoid tissue in the Waldeyer’s lymphatic ring [34,69,70]. The combined functional (PET) and CT examination has an inability to identify small tumors under 5 to 10 mm in diameter [17,71], which is a striking issue in the event of SCCUP diagnostics where identified primary sites are reported with a mean size of 0.78–1.3 cm [12,72,73,74].

In OPSCC, where the tumor is successfully visualized on CT scans, it is hypothesized that the modality is able to distinguish between HPV− and HPV+. This is because HPV− tumors are significantly more often invading the adjacent pharyngeal musculature than HPV+ tumors [75], and HPV+ tumors are more often seen as exophytic tumors with clearly defined borders, although this is not statistically significant [75]. The HPV+ cases were, however, significantly more likely to exhibit cystic nodal metastases than HPV− tumors [75], which can be translated to SCCUP since cystic morphology is a common trait in both SCCUP and OPSCC [59]. Recently, in a hypothesis-generating study, a deep learning algorithm of PET-images was able to successfully distinguish between HPV+ and HPV− OPSCC disease with an AUC of 0.83 [76]. Future radiological imaging in SCCUP will most likely benefit from possible deep learning algorithms. Transcervical ultrasound has also been introduced in the diagnostic workup, and in an unblinded prospective cohort study including 51 patients with suspected or confirmed OPSCC, it correctly identified 90.2% of the tumors and identified 10/14 tumors that were missed by CT [77]. However, with *n* = 47 being p16+, the study unfortunately did not reveal how many of the patients were classified as SCCUP before enrolment, but the average dimension of identified tumors was 2.3 cm [77].

### 3.3. Surgical Procedures

After radiological imaging and clinical examination, the patient is normally scheduled for an examination under general anesthesia (pan-endoscopy). The identification rate is 22–31% after direct pharyngo-laryngoscopy [9,78,79], 28–30% after palatine tonsillectomy [9,80,81], and 18% after base of the tongue biopsies [82]. It is noteworthy that the cumulative identification rate differs remarkedly in HPV− and HPV+ patients, with reported cumulative identification rates of 26% versus 65%, respectively [9].

An addition to the clinical examination of SCCUP patients in the pursuit of successful primary tumor site identification is narrow band imaging (NBI). The endoscopic tool can visualize neo-angiogenic formations that are characteristic of malignant tumors by restricted light wavelength. In a recent meta-analysis comprising SCCUP patients, the use of NBI showed a sensitivity and specificity of 83% and 88%, respectively, and the overall identification rate was 35%, disregarding HPV status which was not reported [83].

In 2009, the FDA approved the use of TORS for early-stage OPSCC [84]. The procedure has, correspondent to the surge in HPV-induced OPSCC, been increasingly adopted in surgical oncological treatment, with an increase of 67% in the United States in the early 2010s, especially in the treatment of HPV+ cancer [85]. The use of TORS in HPV+ disease was further shown to be associated with reduced likelihood of adjuvant CRT versus adjuvant radiotherapy, and the risk of positive margins was significantly reduced when comparing TORS with conventional surgery [85].

The utility of TORS and TLM-assisted lingual mucosectomy has also been proven as a new diagnostic modality for SCCUP patients, with successful identification rates of 70–78% of the primary tumor sites [34,86,87,88]. These diagnostic modalities provide improved visualization and access to an anatomically complex area, and freedom of movement compared to conventional surgical techniques when resecting the superficial part of the tongue base [34], and are therefore advancing as essential diagnostic tools in SCCUP. The procedure is, as also proven in previous diagnostic steps, highly effective in identifying the primary tumor site in the group of patients with HPV+ lymph node metastasis, as 55–96% of the identified tumors are HPV+ [87,88]. This is contrasted by an identification rate of only 13% in HPV− cases [89]. The procedure consequently entails improved tumor detection in HPV+ cases with the potential for radical surgical treatment when the tumor site is identified. Even when there is an indication for adjuvant medical oncological treatment, the identification of the primary tumor site effectuates a notable volume reduction in the adjuvant RT and hence de-escalates oncological treatment [9,90,91].

Synchronous tumors have been demonstrated to occur in 6–18% of palatine and lingual tonsil cancers [9,64,80,81,91,92]. The rate of synchronous cancers in HPV+ cases is reported by two studies, one of which found a rate of 7.7% in HPV+ patients with SCCUP [91], while a second found a rate of 5% among SCCUP patients where 92% of the synchronous tumors were HPV+, with the highest incidence in the mid-2000s and early 2010s [92]. Nevertheless, the literature regarding the frequency of synchronous tumors conflicts with other studies reporting a lower risk of synchronous primary tumors with p16+ [93] and an incidence of synchronous or metachronous OPSCC between 0.5% and 2.5% [94]. However, the authors found a prevalence from 1% to 10% when pooling patients with both HPV+ and unknown HPV status [94]. Given the lack of evidence and conflicting prevalence, a bilateral mucosectomy approach should strongly be considered due to the high rate, between 2% and 15%, of contralateral disease in the palatine and lingual tonsils in studies with 71–82% HPV+ cases [34,86,95].

## 4. Treatment

Referring to a European multi-center study, HPV+ SCCUP generally possesses a better prognosis than its HPV− counterparts, with significantly better overall survival and progression-free survival [32]. A Swedish cohort study also found an increased 2-, 5-, and 10-year survival of 93%, 88%, and 82%, respectively, for p16+ SCCUP versus a 67%, 61%, and 39% 2-, 5- and 10-year survival, respectively, for p16- SCCUP [96]. A recent meta-analysis of SCCUP also found a better survival in HPV+ disease, with 5-year overall survival of 91% compared to 44% overall survival with HPV− disease [19]. This improved survival is in accordance with the significantly better survival recorded in OPSCC disease, especially in the case of both HPV+ and p16+ [35,97].

The identification of the primary tumor site is mandated for an optimal treatment.

A retrospective study (*n* = 136) has highlighted the importance of identifying the primary tumor site as this leads to improved overall survival, cause-specific survival, and disease-free survival, also when stratified for HPV status [98]. The adjuvant RT is altered for targeted therapy with a reduced RT volume and RT field after identification of the primary [9,90,91,99]. In retrospective studies with 92–100% HPV+ cohorts, it is demonstrated that between 25% and 30% of the patients can be treated solely with TLM or TORS [72,100,101,102]. In one cohort study including p16+ patients and another including >66% HPV+ patients, it has been proposed that the pharyngeal mucosa can be sparred in 44–50% of the patients [91,102], thereby limiting multimodal therapy. Even after a failed primary tumor site identification, there is no difference in overall, local and distant, or regional recurrence-free survival when omitting the pharyngeal mucosa in the RT field for p16+ patients after a thorough diagnostic workup including TORS with clearance of the lymphoid tissue in the oropharynx [103]. The de-escalation strategy significantly limited grade 2+ mucositis, opioid treatment, weight loss, feeding tube placement, and unplanned hospital admissions [103].

Recent literature also suggests that ECE in the case of HPV+ does not signify the same risk of an inferior treatment outcome as its HPV− counterpart. A recent study (*n* = 82) from Ireland has demonstrated that ECE in association with p16+ does not significantly influence recurrence-free survival and disease-free survival when the majority of patients received adjuvant RT [104], and the use of adjuvant CRT therapy might, therefore, be questioned. Similar results have also been demonstrated among p16+ OPSCC patients [105,106].

Furthermore, a retrospective database study from the Commission on Cancer National Cancer Database in the United States (*n* = 978) demonstrated that cN2 and cN3 HPV+ patients had similar survival regardless of treatment choice, be that surgery, surgery with adjuvant RT, or surgery with adjuvant CRT or definitive CRT or definitive RT on multivariate analysis, while de-escalation therapy in HPV− patients was associated with worse outcome [107].

De-escalated therapy may therefore be an option selected for HPV+ SCCUP. However, the efficacy assessment of a de-escalation therapy strategy requires long follow-up periods and is still awaited.

## 5. Discussion

The risk of HPV infection during a lifetime is striking, as most sexually active persons will acquire the infection at some point [108]. The HPV infection can be cleared and only exist transiently, but if persistent, it can be oncogenic [109], with subsequent risk of developing OPSCC [42].

The frequency of SCCUP has significantly increased in recent decades, as illustrated by a single-institution study from the United States in which the mean number of SCCUP cases increased from 3.5 per year in 2005–2008 to 15.6 per year in 2012–2014, in parallel with optimized diagnostic primary tumor-detection rates after implantation of TORS [5]. A large Danish cohort study also observed a significant rise in palatine tonsil and base of tongue cancers from 2000 to 2017, and the surge in base of tongue cancers is speculated to be originating from increased use of TORS tongue-base mucosectomy in patients with SCCUP [6].

HPV status has been added to the latest 8th edition of the AJCC cancer staging manual, since there is a distinct difference between HPV+ and HPV− cases and the prognosis. The superior survival, especially in HPV+ and p16+ OPSCC patients, makes this patient category particularly suitable for de-escalation trials [35]. The distinction between HPV+ and HPV− patients has also been shown to reflect the patient’s comorbidity burden. From a large population-based study, it is demonstrated that HPV+ patients had significantly fewer comorbidities, both at the time of diagnosis and throughout the subsequent treatment, compared to HPV− patients, and that HPV− patients also had a higher occurrence of multiple comorbidities, e.g., cerebrovascular disease, other malignancy, and dementia [110]. This is also evident in a study where, e.g., cerebrovascular disease was significantly lower among patients with HPV+ disease [111]. For this reason, it is mandatory to determine the correct HPV status of the lymph node metastasis due to the potential implications it harbors for guidance in diagnostics and treatment, as this can lead to a more personalized paradigm in favor of the patients, clinicians, and healthcare system.

Failure to identify the primary tumor despite extensive diagnostic workups may reflect the small tumor size located deep in the tonsillar crypts, or it may be a question of an immune-mediated response, as the immune system has been demonstrated to be active in combatting HPV+ OPSCC [28,37,44,46,48,49]. A key point in the identification of the primary tumor is the thorough histological examination of the resected specimen, whether palatine or lingual tonsils, performed by the pathologist. An ongoing study aims to evaluate the feasibility and identification-rate of step serial sectioning versus conventional histology of the resected tongue base biopsy and palatine tonsils, with the hope of increased detection rates of the primary tumor site that would otherwise have been missed by the conventional histological technique (NCT04151134).

Given the better prognosis and possibility of de-escalation therapy with HPV+, several ongoing trials are aiming to tailor and reduce RT (NCT04489212, NCT02764216, NCT03323463). However, the relatively few annual SCCUP patients and the diverse diagnostic and treatment modalities used across institutions are limiting the possibilities of a multi-center, multi-national, and randomized clinical trial.

The addition of TORS and TLM as a new diagnostic and treatment modality has proven a useful and highly effective tool in the search for the primary tumor. In several trials, the identification rate has increased with the addition of TORS and TLM-assisted lingual mucosectomy compared to traditional diagnostic approaches [5,9,34,112]. The TORS procedure also allows for complete surgical, oncologic lingual mucosectomy with negative margins in 60–80% of the cases [87,88]. One ongoing study from Canada is pursuing the successful identification of SCCUP with TORS among T0, N1-N3, and M0 patients and possible de-intensified adjuvant RT therapy (NCT03281499) but could be limited by its small sample size (*n* = 22) and recruitment not limited by HPV status, yet similar designs should be implemented as a research purpose at multiple institutions. TORS has been proven as a safe procedure with limited effect on QoL [34,113,114]; however, one of the primary reasons for unplanned readmission following TORS is pain [115]. This is being investigated in an ongoing Danish national randomized clinical trial assigning patients to either a high-dose or low-dose dexamethasone treatment regime following TORS (NCT04189107) [116]. Future research should assess QoL and functional outcomes when comparing treatment approaches. In OPSCC, this is applied in the comparison of TORS and CRT, e.g., the ongoing Danish randomized clinical trial (NCT04124198, quality of life after primary TORS vs IMRT “The QoLATI study” DAHANCA-34). These QoL and functional evaluations combined with patient-related but objective measurements such as days alive and out of hospital, which is a patient-related outcome metric that reflects the burden of a given disease but also the treatment offered [116], should be implemented in upcoming treatment-comparing studies.

In conclusion, HPV+ SCCUP is a challenging disease entity for patients, clinicians, and researchers with a lack of consensus regarding diagnostic procedures, treatment, and understanding of the pathophysiology in disease development. Fortunately, awareness is increasing as indicated by the recent 8th AJCC cancer staging manual classifying SCCUP based on HPV status rather than a uniform disease entity, as it was considered up until recently. Different diagnostic workup algorithms and treatment options should also be based on HPV status. The HPV-induced SCCUP disease could be seen as a subgroup of OPSCC, but with a tumor so small that it is missed during radiological imaging and histological examinations of resected specimens, supported by the observed increase in identification rates with the implementation of advanced modalities as TORS, TLM, and PET-CT. Several ongoing trials are aiming at optimizing diagnostics and treatment modalities, but given the rarity of the condition, the advancement must, so far, be reliant on retrospective and prospective cohort studies.
